# Vaginal fungi are associated with treatment-induced shifts in the vaginal microbiota and with a distinct genital immune profile

**DOI:** 10.1128/spectrum.03501-23

**Published:** 2024-06-24

**Authors:** Eric Armstrong, Anke Hemmerling, Steve Miller, Sanja Huibner, Maria Kulikova, Rachel Liu, Emily Crawford, Gloria R. Castañeda, Bryan Coburn, Craig R. Cohen, Rupert Kaul

**Affiliations:** 1 Department of Medicine, University of Toronto, Toronto, Canada; 2 Department of Obstetrics, Gynecology & Reproductive Sciences, University of California, San Francisco, San Francisco, California, USA; 3 Department of Laboratory Medicine, University of California, San Francisco, San Francisco, California, USA; 4 Delve Bio, San Francisco, California, USA; 5 Toronto General Hospital Research Institute, University Health Network, Toronto, Canada; 6 Department of Microbiology and Immunology, University of California, San Francisco, San Francisco, California, USA; 7 Chan Zuckerberg Biohub, San Francisco, California, USA; 8 Department of Medicine, University Health Network, Toronto, Canada; Wayne State University, Detroit, Michigan, USA

**Keywords:** genital immunology, vaginal microbiome, fungi, *Candida*, inflammation

## Abstract

Vaginal colonization by fungi may elicit genital inflammation and enhance the risk of adverse reproductive health outcomes, such as HIV acquisition. Cross-sectional studies have linked fungi with an absence of bacterial vaginosis (BV), but it is unclear whether shifts in vaginal bacteria alter the abundance of vaginal fungi. Vaginal swabs collected following topical metronidazole treatment for BV during the phase 2b, placebo-controlled trial of LACTIN-V, a *Lactobacillus crispatus*-based live biotherapeutic, were assayed with semi-quantitative PCR for the relative quantitation of fungi and key bacterial species and multiplex immunoassay for immune factors. Vaginal fungi increased immediately following metronidazole treatment for BV (adjusted *P* = 0.0006), with most of this increase attributable to *Candida albicans*. Vaginal fungi were independently linked to elevated levels of the proinflammatory cytokine interleukin (IL) 17A, although this association did not remain significant after correcting for multiple comparisons. Fungal relative abundance by semi-quantitative PCR returned to baseline levels within 1 month of metronidazole treatment and was not affected by LACTIN-V or placebo administration. Fungal abundance was positively associated with *Lactobacillus* species, negatively associated with BV-associated bacteria, and positively associated with a variety of proinflammatory cytokines and chemokines, including IL-17A, during and after study product administration. Antibiotic treatment for BV resulted in a transient expanded abundance of vaginal fungi in a subset of women which was unaffected by subsequent administration of LACTIN-V. Vaginal fungi were positively associated with *Lactobacillus* species and IL-17A and negatively associated with BV-associated bacteria; these associations were most pronounced in the longer-term outcomes.

**IMPORTANCE:**

Vaginal colonization by fungi can enhance the risk of adverse reproductive health outcomes and HIV acquisition, potentially by eliciting genital mucosal inflammation. We show that standard antibiotic treatment for bacterial vaginosis (BV) results in a transient increase in the absolute abundance of vaginal fungi, most of which was identified as *Candida albicans*. Vaginal fungi were positively associated with proinflammatory immune factors and negatively associated with BV-associated bacteria. These findings improve our understanding of how shifts in the bacterial composition of the vaginal microbiota may enhance proliferation by proinflammatory vaginal fungi, which may have important implications for risk of adverse reproductive health outcomes among women.

## INTRODUCTION

The vaginal microbiota is an ecosystem comprised of microbes from a variety of taxonomic groups, including bacteria, fungi, and viruses, and its composition is associated with reproductive health (1). The bacterial component of the vaginal microbiota is generally dominated either by *Lactobacillus* species or by a diverse population of bacteria, with the latter defined as bacterial vaginosis (BV) (2). Although less well-characterized vaginal fungi typically belong to the genus *Candida*, with *Candida albicans* being most common, and can frequently be observed among women without symptoms of a fungal infection (3). Although fungal overgrowth can cause symptomatic vulvovaginal candidiasis (4), even asymptomatic colonization by vaginal fungi appears to enhance the risk of adverse reproductive health outcomes, such as HIV acquisition and preterm birth (5, 6).

Vaginal fungi and the bacterial composition of the vaginal microbiota have been closely linked in cross-sectional studies, with the absence of BV and presence of vaginal *Lactobacillus* species linked to an increased detection of vaginal *C. albicans* (7, 8). Although the longitudinal dynamics of bacterial and fungal interaction in the female genital tract has not been characterized, the increased frequency of vulvovaginal candidiasis observed after antibiotic treatment of BV suggests that perturbations in the bacterial microbiota may enhance fungal proliferation (9, 10). However, there is little information on how fungi respond to specific changes in the bacterial composition of the vaginal microbiota and the impact of such responses on genital immunology. Here, we characterize vaginal fungal dynamics in a subset of women from the randomized, placebo-controlled phase 2b trial of LACTIN-V, a *Lactobacillus crispatus*-based live biotherapeutic, following metronidazole treatment for BV (11).

## RESULTS

### Antibiotic treatment for BV increases the abundance of vaginal fungi

**Fig 1:**
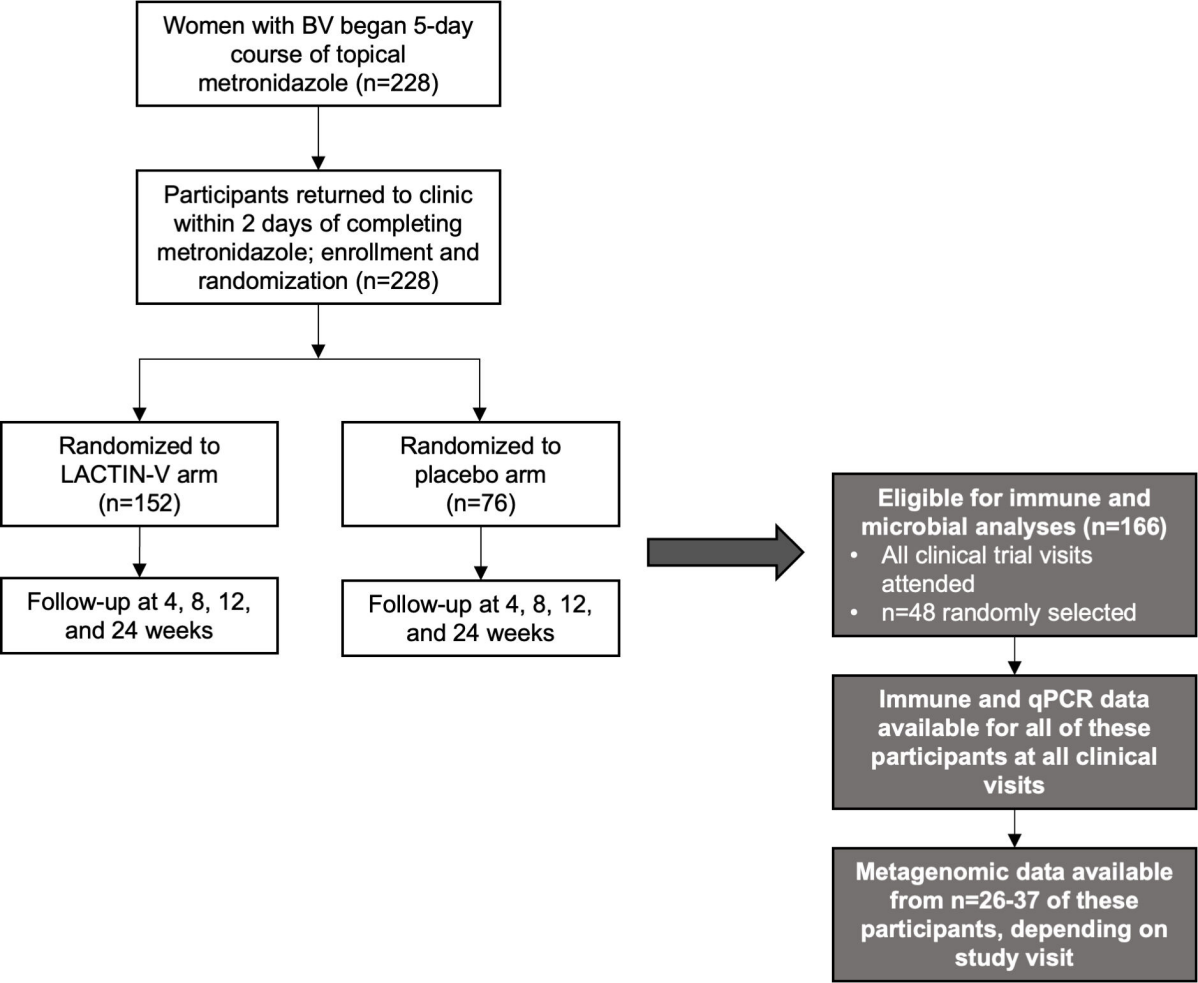
Analysis of fungal dynamics in the LACTIN-V clinical trial. Study design diagram amended from the LACTIN-V clinical trial (11) and a previous analysis of this subset of participants (12).

**Fig 2:**
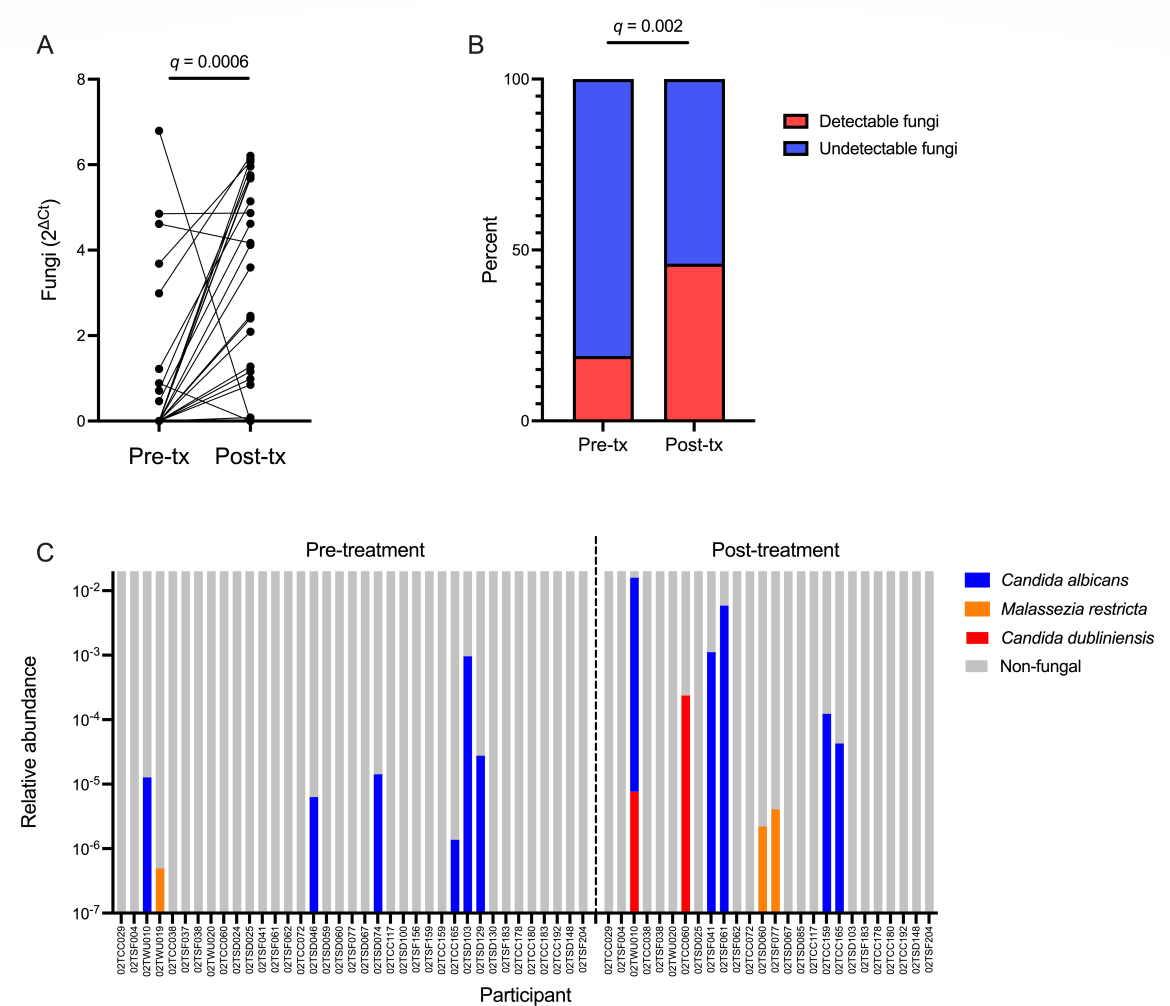
Vaginal fungal dynamics during metronidazole treatment. (**A**) Change in fungal abundance immediately following metronidazole treatment. (**B**) Proportion of women with detectable fungi by semi-quantitative PCR before and immediately after metronidazole treatment. (**C**) Species-level classification of vaginal fungi identified with metagenomic sequencing immediately before and after metronidazole treatment expressed as a proportion of total non-host reads. Participant ID annotated beneath x-axis. Paired comparison performed with Wilcoxon matched pairs signed-rank test. *P*-values adjusted for multiple comparisons with the false discovery rate (FDR). FDR-corrected *q* values presented on figure.

Study design of the larger LACTIN-V trial and the present sub-analysis is presented in Fig. 1. Vaginal fungi were detectable by semi-quantitative PCR (defined as a relative quantity greater than 0) in 9 of 48 (19%) participants with BV prior to BV treatment and in 22 of 48 (46%) participants immediately following metronidazole treatment (*q* = 0.002; Fig. 2).

The relative quantity of vaginal fungi compared to negative controls by semi-quantitative PCR significantly increased immediately following metronidazole treatment (*q* = 0.0006; Fig. 2). Upon establishing a clear change in fungal abundance following metronidazole treatment, we conducted exploratory analyses to assess the taxonomic composition of these fungi and correlates of fungal expansion. In a subset of these participants from whom metagenomic sequencing data were available, fungal reads were identified among 7 of 37 (19%) participants prior to metronidazole and 8 of 26 (31%) immediately following metronidazole. Fungi comprised a small proportion of total non-host reads and were primarily classified as *C. albicans*, although *Malassezia restricta* and *Candida dubliniensis* were also identified (Fig. 2). Fungal detectability was concordant between semi-quantitative PCR and metagenomic sequencing at pre- and post-metronidazole visits (*P* = 0.015 and *P* = 0.003, respectively, with Fisher’s exact test; Table S2). To assess concordance in fungal quantitation between our semi-quantitative PCR and metagenomic results, we also tested the correlation between the proportion of fungal to bacterial abundance (as measured by semi-quantitative PCR) and the proportional abundance of all fungal reads relative to total non-host reads (as measured by metagenomic sequencing) before and after metronidazole treatment. The proportion of fungal to bacterial abundance by semi-quantitative PCR was positively correlated with the proportion of fungal to total non-host reads by metagenomic sequencing at the pre-metronidazole (Spearman’s ρ = 0.54, *P* = 0.0005) and post-metronidazole (Spearman’s ρ = 0.63, *P* = 0.0005) visits, although these results should be interpreted with caution given the highly zero-inflated nature of our fungal data. To explore predictors of fungal expansion following metronidazole, we stratified the cohort based on whether fungi were detectable by semi-quantitative PCR at the post-metronidazole visit. Using linear regression, we found that pre- and post-treatment fungal abundance was positively associated (B = 0.51, *P* = 0.024). Similarly, post-treatment detectability of fungi tended to be more prevalent among women who had detectable fungi prior to treatment compared to those who did not (*P* = 0.078; Fig. S1). Detectability of fungi following metronidazole treatment was not associated with sociodemographic factors, sexual activity, or baseline hormonal contraceptive use (Table S3). We also characterized the overall composition of the vaginal microbiota, abundance of key bacterial species, and levels of soluble immune factors prior to metronidazole treatment, but none of these factors differed based on fungal detectability post-treatment (Fig. S2 and S3).

### Fungal detection immediately after BV treatment associated with elevated *Lactobacillus* species and proinflammatory cytokine levels

**Fig 3:**
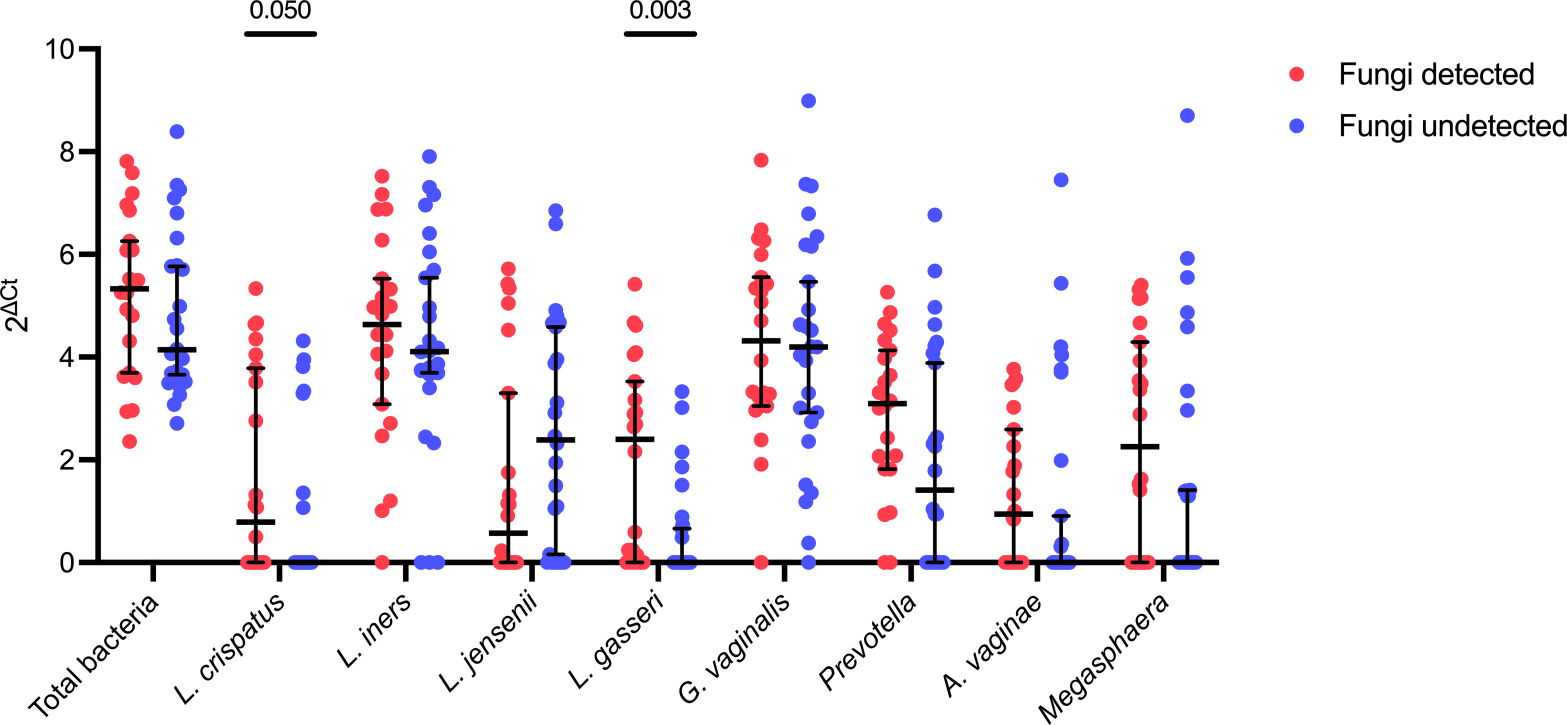
Comparison of bacterial abundances following metronidazole treatment based on fungal detectability. Comparison of total bacterial abundance and abundance of *L. crispatus*, *L. iners*, *L. jensenii*, *L. gasseri*, *G. vaginalis*, *Prevotella*, *A. vaginae*, and *Megasphaera* immediately following metronidazole treatment between women with detectable and undetectable fungi immediately following treatment. Data are median and 95% confidence intervals. *P*-values determined with Mann-Whitney U-test. Only *q* values <0.05 after FDR correction were included in the figure.

**Fig 4:**
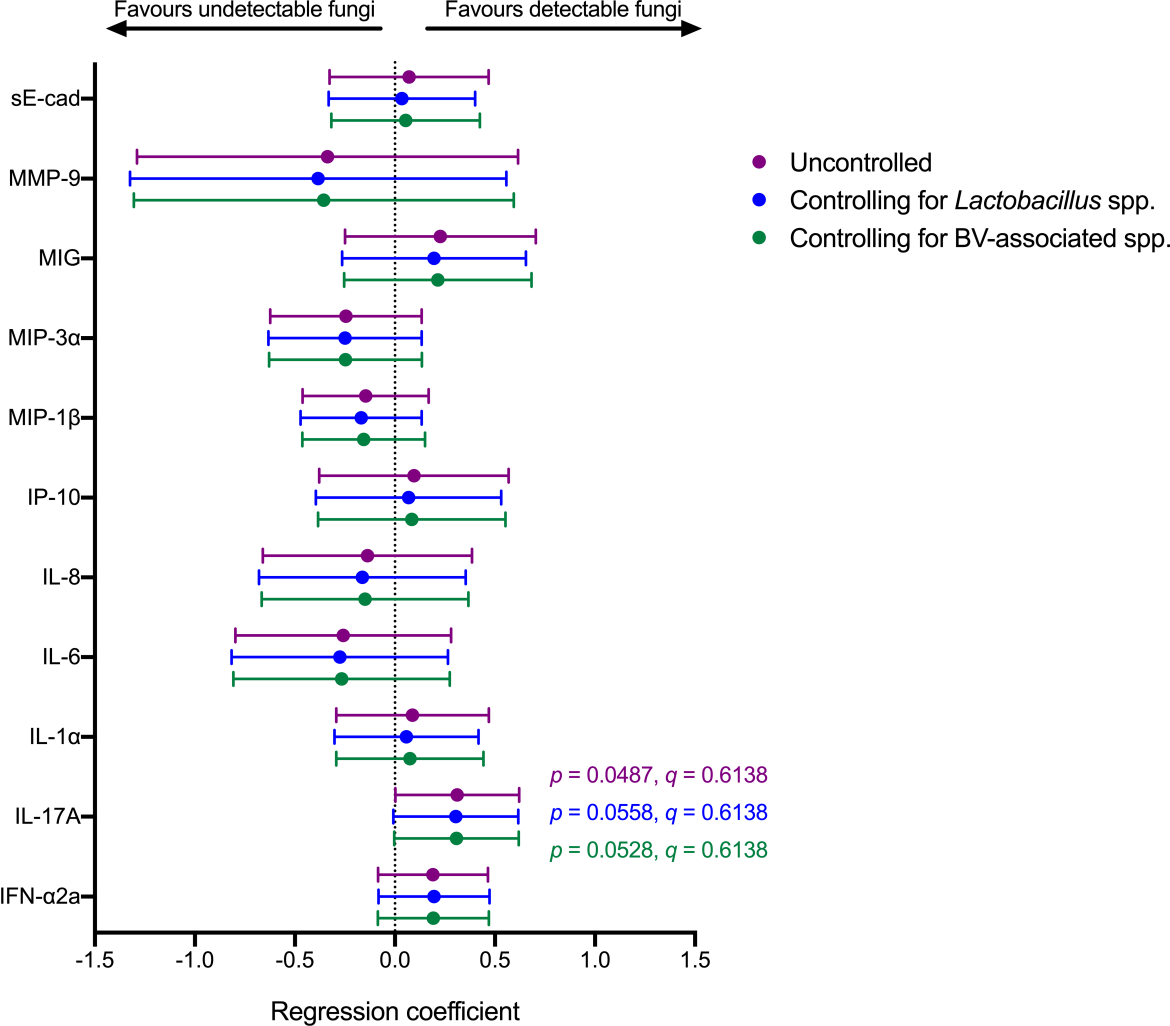
Association between detectable fungi and genital immune factors immediately following metronidazole treatment. Forest plot displaying linear regression coefficients and 95% confidence intervals for linear models measuring the association between fungal detectability and soluble immune factors immediately following metronidazole treatment. Linear models included soluble immune factors as dependent variables and either fungal detectability (purple datapoints), fungal detectability and the summed abundance of *Lactobacillus* spp. (*L. crispatus, L. iners, L. jensenii,* and *L. gasseri*; blue datapoints), or fungal detectability and the summed abundance of BV-associated bacteria (*G. vaginalis, Prevotella* spp.*, A. vaginae,* and *Megasphaera* spp.; green datapoints) as independent variables.

Immediately following metronidazole treatment, the presence of vaginal fungi by semi-quantitative PCR was not associated with differences in overall vaginal microbiota composition [analysis of similarity (ANOSIM) *R* = 0.007, *P* = 0.39; Fig. S4], although there was a trend toward lower Shannon diversity among women with detectable fungi compared to women with undetectable fungi (log fold change = −0.31; *P* = 0.08; Fig. S4). Among women with detectable fungi immediately following metronidazole treatment, post-treatment abundance of *Lactobacillus gasseri* (log fold change = 1.44, *P* = 0.003, *q* = 0.02) and *L. crispatus* (log fold change = 0.87, *P* = 0.05, *q* = 0.22) were higher compared to women that did not have detectable fungi post-treatment, although the latter did not remain significant after false discovery rate (FDR) correction. (Fig. 3). Next, we generated linear regression models to predict post-treatment levels of vaginal soluble immune factors with post-treatment fungal detectability. Fungal detectability immediately following metronidazole was positively associated with vaginal interleukin (IL) 17A levels (B = 0.31, *P* = 0.049, *q* = 0.6138), which did not remain significant after correcting for multiple comparisons. Since the vaginal microbiota is closely related to the genital immune milieu and therefore may confound the relationship between vaginal fungi and genital immune factors such as IL-17A, we also generated linear models that controlled for the abundance of key *Lactobacillus* species or BV-associated bacteria. IL-17A remained positively associated with fungal detectability in models that included the summed abundance of *L. crispatus*, *Lactobacillus iners, Lactobacillus jensenii*, and *L. gasseri* (B = 0.30, *P* = 0.056, *q* = 0.6138) or the summed abundance of *Gardnerella vaginalis, Prevotella, Atopobium vaginae,* and *Megasphaera* (B = 0.31, *P* = 0.053, *q* = 0.6138) as covariates, although these tests did not remain significant after correcting for multiple comparisons (Fig. 4).

### Fungal expansion following metronidazole treatment is transient and unaffected by administration of an *L. crispatus* live biotherapeutic

**Fig 5:**
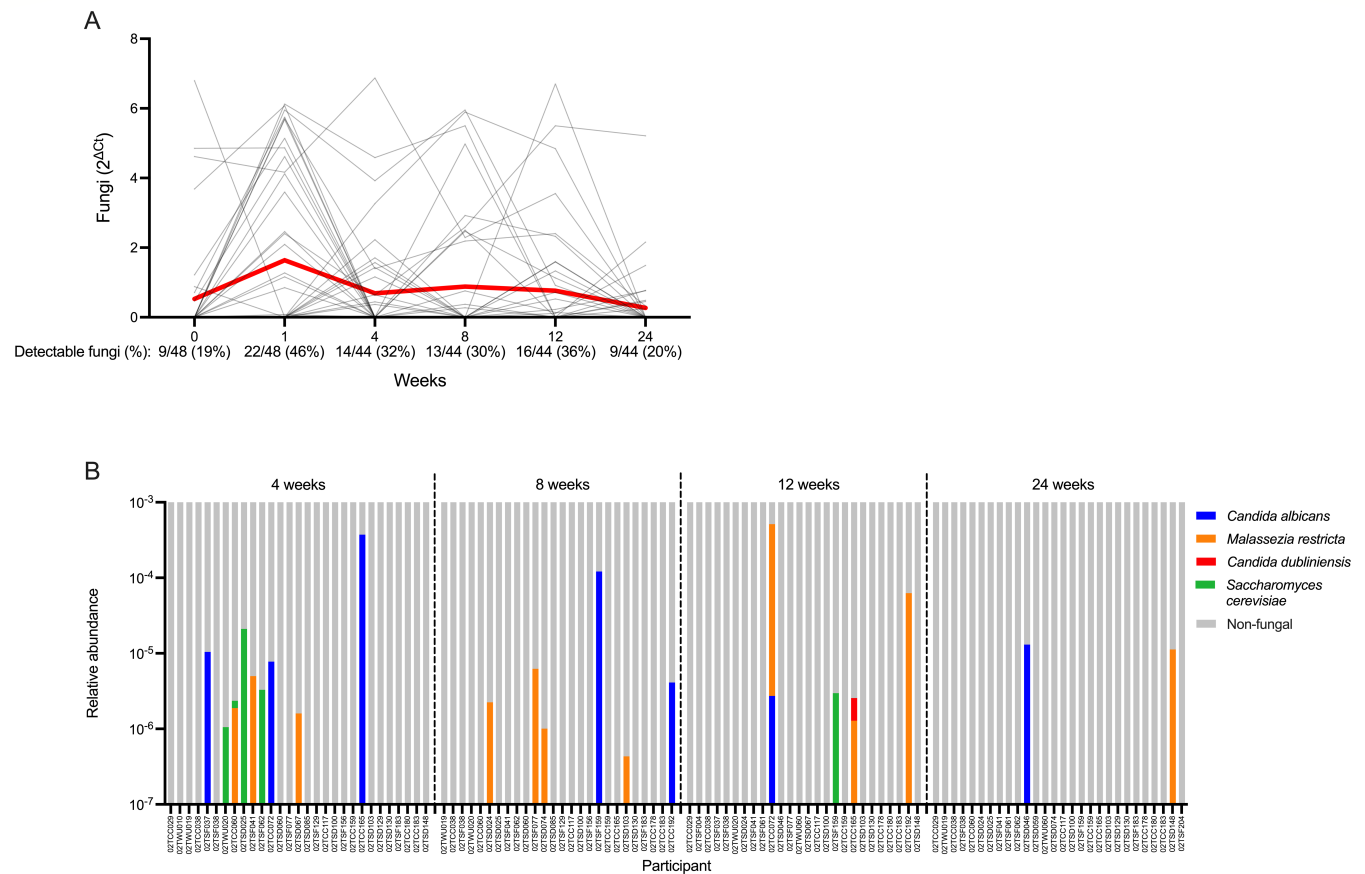
Vaginal fungal dynamics before, during, and after study product administration. (**A**) Comparison of baseline fungal abundance to each visit during and after study product administration. Paired individual values shown in gray and mean shown in red. *P*-values calculated with Wilcoxon matched pairs signed-rank test. No significant associations or trends were observed before correcting for multiple comparisons, and so no *P*-values are presented in the figure. Fungal detectability during and after study product administration annotated below x-axis. (**B**) Relative abundance of fungal species identified with metagenomic sequencing during and after study product administration expressed as a proportion of total non-host reads. Participant ID annotated beneath x-axis.

**Fig 6:**
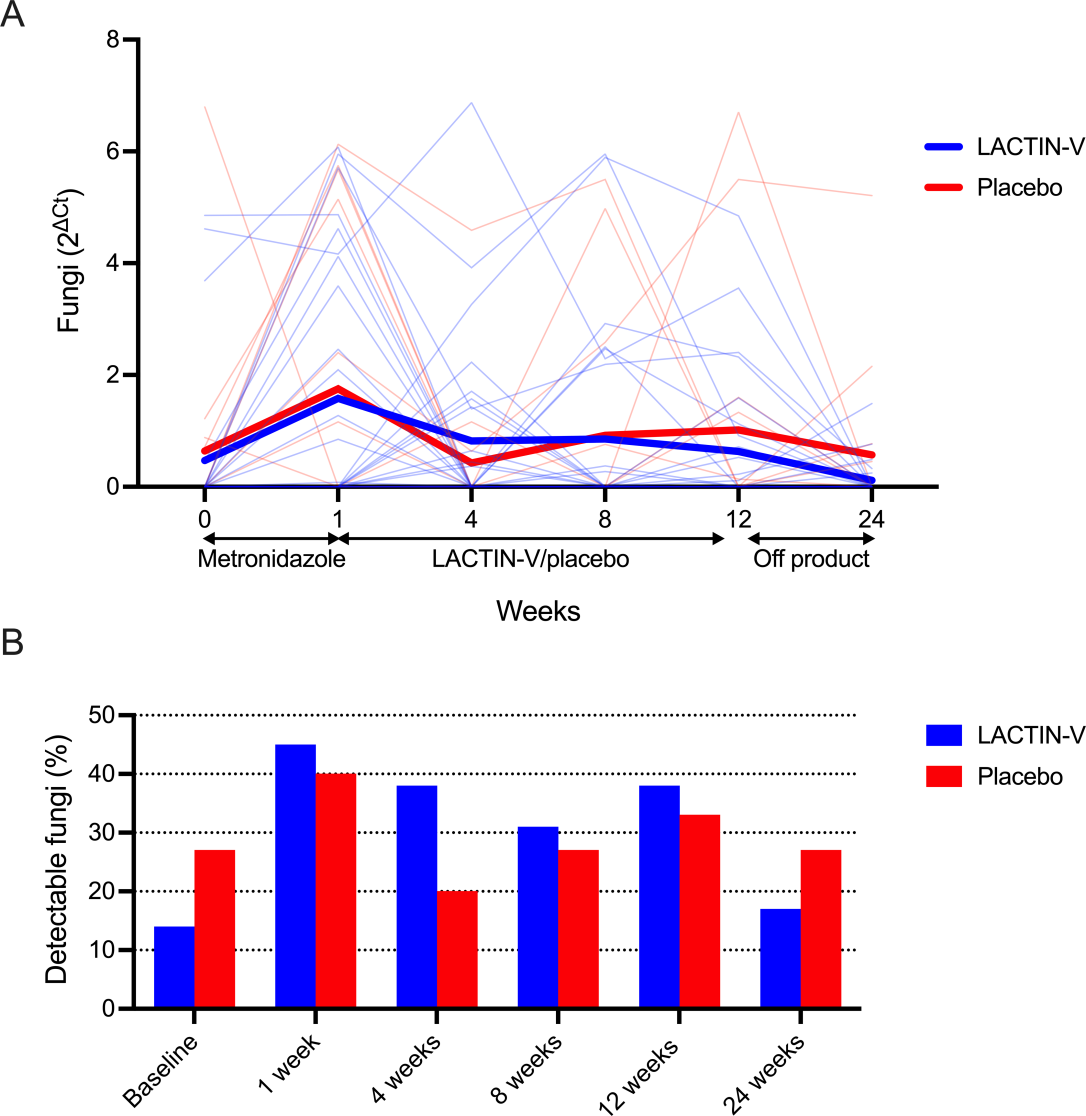
Impact of LACTIN-V on vaginal fungi. Fungal (**A**) abundance and (**B**) detectability at each visit based on LACTIN-V or placebo receipt. Comparisons performed with the Mann-Whitney U-test or Pearson’s χ^2^ test. No significant associations or trends were observed before correcting for multiple comparisons, and so no *P*-values are presented in the figure.

Following metronidazole treatment, all participants were randomized to receive either the *L. crispatus*-based live biotherapeutic LACTIN-V or a matched placebo, each of which was self-administered intravaginally for 11 weeks. Four participants were diagnosed and treated for symptomatic yeast vaginitis during or after LACTIN-V or placebo administration: three in the LACTIN-V group and one in the placebo group. These participants were included in the previous analysis of samples collected immediately before and after metronidazole treatment but were excluded from all subsequent analyses to limit potential confounding by antifungal treatment. Within 4 weeks of completing metronidazole treatment, fungal abundance returned to baseline levels and was relatively unchanged for the remainder of the study, although there was considerable variation in fungal abundance over the course of the study (Fig. 5). During and after LACTIN-V or placebo administration, the vaginal mycobiota was comprised of either *C. albicans, C. dubliniensis, M. restricta*, or *Saccharomyces cerevisiae*, albeit as a small proportion of total non-host reads (Fig. 5). Neither fungal abundance nor detectability by semi-quantitative PCR differed at any timepoint between women who received LACTIN-V and placebo (Fig. 6).

### Fungi are associated with key vaginal bacterial species and immune factors during and after study product administration

**Fig 7:**
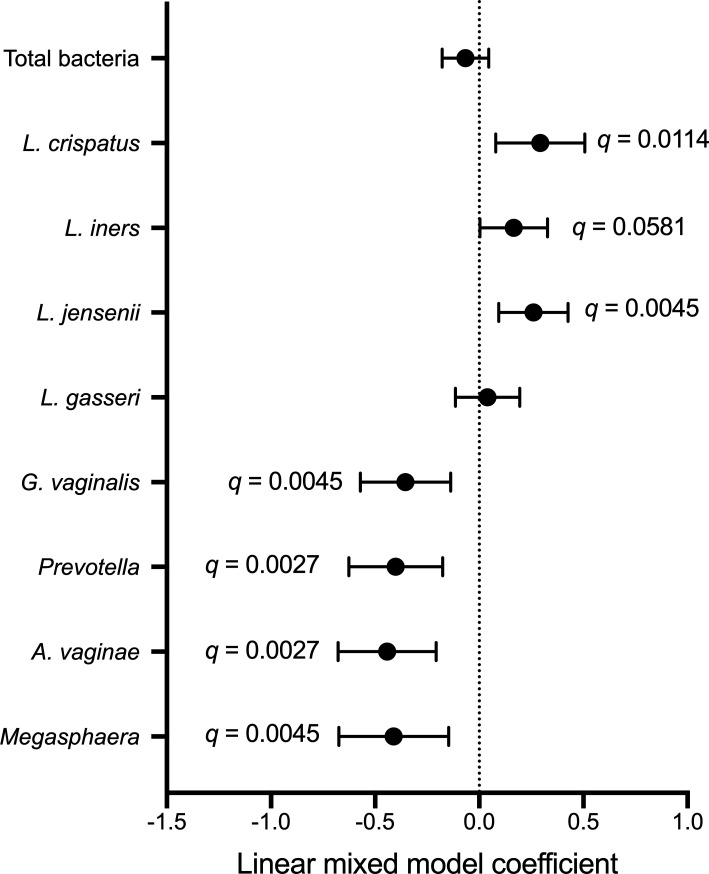
Microbial correlates of fungi during and after study product administration. *P*-values generated with linear mixed models that included timepoints during and after study product administration. FDR-corrected *q* values that remained significant were included in the figure as annotations. Each model included fungal abundance as the independent variable and bacterial abundance as the dependent variable.

**Fig 8:**
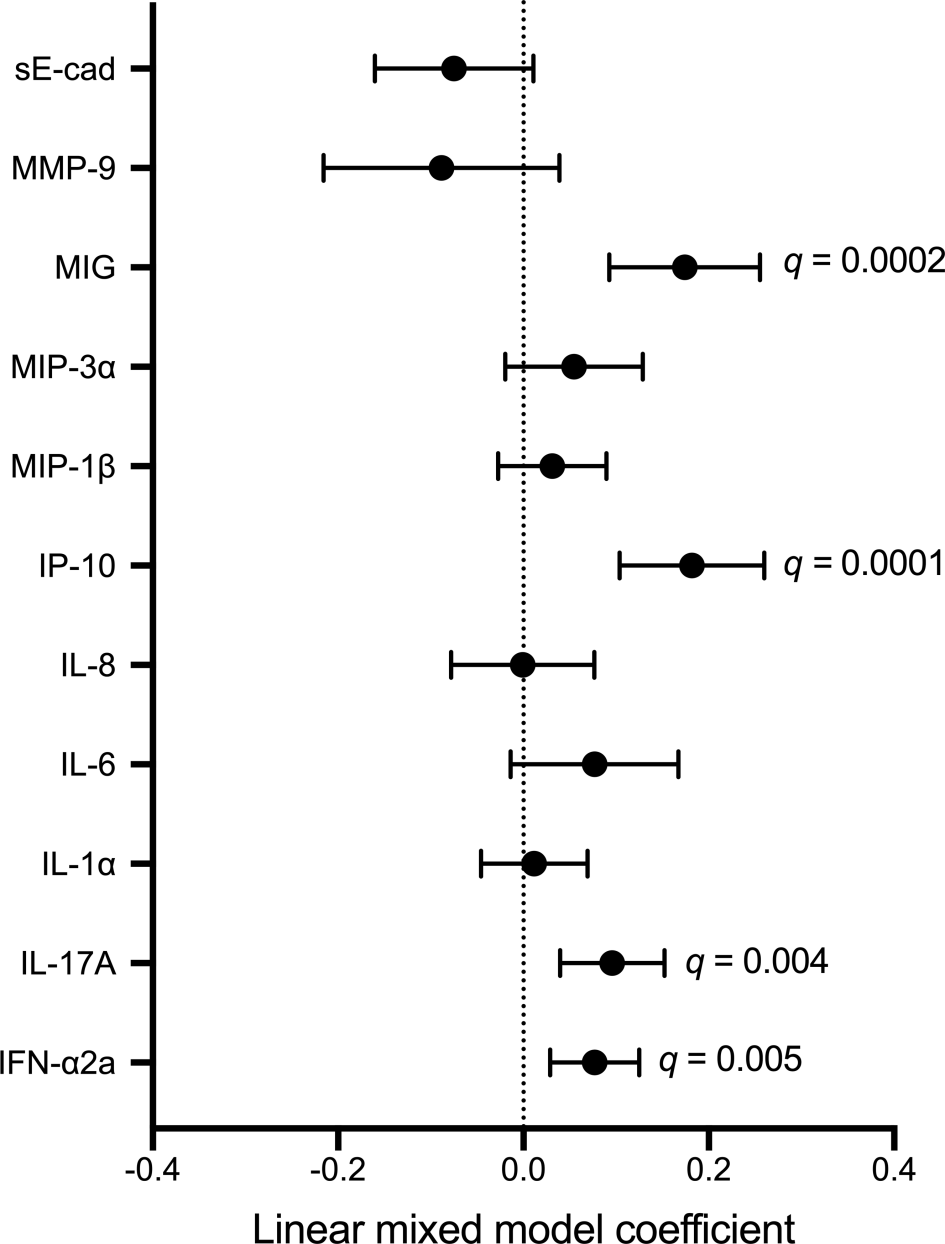
Immune correlates of fungi during and after study product administration. *P*-values generated with linear mixed models that included timepoints during and after study product administration. FDR-corrected *q* values that remained significant were included on the figure as annotations. Each model included fungal abundance as the independent variable and bacterial abundance as the dependent variable.

Next, we explored the microbial and immune correlates of fungal detection and abundance by semi-quantitative PCR at subsequent longitudinal visits. In linear mixed models that included measurements obtained during and after study product administration, irrespective of treatment arm, fungal abundance was positively associated with the abundance of *L. crispatus* (B = 0.29, *q* = 0.0114)*, L. iners* (B = 0.17, *q* = 0.0581), and *L. jensenii* (B = 0.26, *q* = 0.0045)*,* and was negatively associated with abundance of *G. vaginalis* (B = −0.35, *q* = 0.0045)*, Prevotella* spp. (B = −0.40, *q* = 0.0027)*, A. vaginae* (B = −0.44, *q* = 0.0027)*,* and *Megasphaera* (B = −0.41, *q* = 0.0045; Fig. 7). These associations remained largely unchanged after controlling for treatment arm (Table S4). To visualize the longitudinal relationship between vaginal fungi and bacteria, we plotted bacterial abundances at each visit during and after study product administration based on fungal detectability (Fig. S5). Fungal abundance tended to be higher among participants who did not have BV (defined as Nugent score ≥7) at each timepoint during and after study product administration (Fig. S6). Next, we generated linear mixed models to evaluate immune correlates of fungal abundance during and after study product administration. Fungal abundance was positively associated with IL-17A (B = 0.10, *q* = 0.004), interferon (IFN) α2a (B = 0.08, *q* = 0.005), interferon gamma-induced protein (IP) 10 (B = 0.18, *q* = 0.0001), and monokine induced by interferon gamma (MIG) (B = 0.17, *q* = 0.0002; Fig. 8), which remained consistent after controlling for treatment group, *Lactobacillus* species, and BV-associated bacteria. However, fungi were negatively associated with matrix metalloproteinase (MMP) 9 (B = −0.14, *P* = 0.037) and soluble E-cadherin (sE-cad) (B = −0.09, *P* = 0.043) in models controlling for *Lactobacillus* species, and positively associated with IL-6 (B = 0.11, *P* = 0.015) in the model controlling for BV-associated bacteria (Tables S5 to S7). We visualized the relationship between vaginal fungi and immune factors during and after study product administration by plotting levels of immune factors based on fungal detectability (Fig. S7).

## DISCUSSION

Vaginal fungi are an understudied component of the vaginal microbiota and may have important implications for female reproductive health (3). Cross-sectional studies have linked vaginal fungi to a *Lactobacillus*-predominant vaginal microbiota (7), but it is unclear how longitudinal changes in vaginal bacteria might affect fungal detection or abundance. In the current study, we show that topical metronidazole treatment of BV resulted in an expansion of vaginal fungi which was identified as primarily *C. albicans*. Fungal expansion post-metronidazole was positively associated with the abundance of *Lactobacillus* species and elevated levels of the cytokine IL-17A (13). Fungal abundance was unaffected by the subsequent intravaginal administration of an *L. crispatus*-based live biotherapeutic, was positively associated with vaginal levels of proinflammatory cytokines, chemokines, and *Lactobacillus* species, and was negatively associated with BV-associated bacteria.

In a previous analysis of the vaginal microbiota in this cohort, we showed that BV treatment with metronidazole rapidly reduced the abundance of BV-associated bacterial species, while inducing very modest changes in the abundance of *Lactobacillus* species (12). We now show that topical metronidazole treatment also causes an increase in fungal abundance which was positively associated with the abundance of *L. crispatus* and *L. gasseri*. Using metagenomic sequencing, we found that the majority of fungi immediately before and after metronidazole treatment were *C. albicans*, although *M. restricta* and *C. dubliniensis* were also present. The increase in fungal abundance we observed after BV treatment is consistent with previous work that has shown that vaginal fungi are more prevalent among women with a *Lactobacillus*-predominant vaginal microbiota (7). Possible mechanisms underpinning these associations include the production of lactate by *Lactobacillus* species, which can dampen antigen expression on the cell surface of *C. albicans* (14) and modulate host-*C. albicans* immune interactions (15), ultimately facilitating immune evasion and enhanced fungal proliferation. The mechanisms explaining the negative association between BV and vaginal fungi are less clear, but may include competitive inhibition and the production of growth-inhibiting toxins by BV-associated bacteria (8).

Over the course of 6 months after the end of metronidazole treatment, fungal abundance was positively associated with *Lactobacillus* species and negatively associated with BV-associated bacteria and tended to be greater among women who did not have recurrent BV. The taxonomic composition of these fungi was similar to the pre- and post-metronidazole visits, although *S. cerevisiae* was also detected. Altogether, this indicates that vaginal fungi remain positively associated with *Lactobacillus* species and negatively associated with BV-associated bacteria even in the absence of a major perturbation in the microbiota by antibiotics, and strongly suggests a causal relationship between the abundance of vaginal fungi and specific bacteria. Since there were few participants who exhibited symptomatic vaginal yeast infections, we were unable to elucidate whether these associations are also present in the context of symptomatic fungal infections.

The IL-17 family of cytokines, especially IL-17A, plays a key role in anti-fungal defense at mucosal surfaces (13). Recognition of fungi by pattern recognition receptors activates the nuclear factor kappa-light-chain-enhancer of activated B cells (NF-κB) signaling pathway and induces a Th17 response and subsequent IL-17 production through the production of proinflammatory cytokines such as IL-6 (13). The importance of IL-17 in anti-fungal immunity is also exemplified by the elevated risk of candidiasis among individuals with mutations in genes related to IL-17 production (16), impaired IL-17 production (17), and during treatment with IL-17 inhibitors (18). We show that detectability of vaginal fungi immediately after metronidazole treatment for BV is positively associated with vaginal levels of IL-17A, which is consistent with a causative model whereby vaginal fungi elicit IL-17 production. While this association did not remain significant after correcting for multiple comparisons, we also observed a positive association between detectability of vaginal fungi and vaginal levels of IL-17A, IFN-a2a, IL-6, IP-10, and MIG during and after LACTIN-V administration. However, IL-17 was positively associated with vaginal *Lactobacillus* species in the present study and *Lactobacillus* species have been linked to elevated IFN-a2a, IP-10, and MIG (12, 19), suggesting that the relationship between vaginal fungi and these immune factors (excluding IL-17A) may be confounded by *Lactobacillus* species. Therefore, although the association between vaginal fungi and IL-17A levels appeared robust and was observed immediately after metronidazole and during/after study product administration, the link between vaginal fungi and other proinflammatory cytokines and chemokines should be explored in greater detail in future studies to explore possible confounding by bacteria.

This study has several limitations. First, we broadly characterized fungal abundance with semi-quantitative PCR using primer and probe sequences targeting the 18S rRNA gene and could only characterize fungal taxonomy using metagenomic sequencing in a small number of participants. The small number of participants with detectable fungi by metagenomic sequencing limited our ability to evaluate whether the immune and bacterial associations with fungal abundance differed by fungal taxon. We encourage future studies to employ more sensitive methods of fungal characterization such as internal transcribed spacer sequencing to evaluate whether the bacterial and immune associations differ based on fungal species or strains. Second, we did not evaluate fungal gene expression or morphology of vaginal fungi in this study. Fungal morphology and gene expression can be altered by the presence of bacteria and may have implications for fungal virulence (20, 21). Future studies should explore whether the morphology and gene expression of vaginal fungi are affected by shifts in the bacterial microbiota and if this has implications for genital immunology. Third, only four participants were clinically diagnosed with vulvovaginal candidiasis over the course of the study. This small sample size means that we were not able to explore vaginal dynamics of fungi, bacteria, and immune factors among women with this condition. Future studies should explore whether our findings differ during symptomatic or asymptomatic vulvovaginal candidiasis. Although all vaginal swabs were collected using standardized methods, vaginal swabs may sample differing amounts of vaginal secretions which can affect downstream analyses. While sampling depth was not normalized in our study, most of our findings are unlikely to be affected by differences in sampling depth given their magnitude (e.g., several log fold in many cases). Lastly, we calculated fungal and bacterial abundance with semi-quantitative PCR by taking the difference in cycle threshold (Ct) between a sample and negative control. While this method has been validated as an accurate estimate of the absolute copy number of a gene in a previous analysis of this cohort (12), it assumes 100% reaction efficiency and should be interpreted as the relative quantity of a gene relative to a negative control.

In summary, we find that standard antibiotic treatment for BV increased the abundance of vaginal fungi in a subset of women. *C. albicans* was responsible for most of this increase in fungal abundance, which was accompanied by an increase in the abundance of *Lactobacillus* species and the cytokine IL-17A, although the latter did not remain significant after correcting for multiple comparisons. In the 6 months following the end of antibiotic treatment, vaginal fungal abundance returned to baseline levels, was unaffected by randomization to LACTIN-V or placebo groups, and was positively associated with *Lactobacillus* species, several cytokines, and chemokines including IL-17A, and negatively associated with BV-associated bacteria. Understanding the role fungi play in the vaginal microbiota may help to elucidate their genital immune effects and impact on adverse reproductive health outcomes.

## MATERIALS AND METHODS

### Study participants

Participants were recruited as part of a phase 2b, randomized, placebo-controlled clinical trial of the *L. crispatus*-based live biotherapeutic LACTIN-V to prevent BV recurrence following topical metronidazole treatment (NCT02766023; May 2016) (11). Two hundred twenty-eight women were enrolled in the larger clinical trial and followed over 6 months, and a subset of 48 participants who attended all clinical trial visits were selected for the current sub-study. Details about the clinical trial and this subset of participants have been published in detail elsewhere (11, 12). Briefly, women with BV (defined by ≥3 Amsel criteria and a Nugent score ≥4) were provided with a 5-day course of vaginal metronidazole and randomized 2:1 to either LACTIN-V or placebo, which were applied vaginally once daily for 5 days followed by twice weekly for an 10 additional weeks. Vaginal swabs were collected at screening prior to metronidazole treatment, within 48 hours of treatment completion and then at 4, 8, 12, and 24 weeks after the start of metronidazole. Following collection, vaginal swabs were plunged into Starswab Multitrans transport medium and frozen at −20°C or −80°C, depending on study site. Two of the swabs collected at each visit were analyzed here; one swab was analyzed using metagenomic sequencing and the second was analyzed with semi-quantitative PCR and immunoassay. All participants were negative for yeast vaginitis based on wet mount performed at screening before and at enrollment after metronidazole treatment. Participants who subsequently tested positive for yeast vaginitis during study product administration were provided standard treatment and continued to receive study product.

### DNA extraction and semi-quantitative PCR

DNA was extracted from 175 µL of bacterial pellet from vaginal swab samples using the Qiagen DNEasy PowerSoil kit (Qiagen) according to manufacturer’s instructions. Targeted semi-quantitative PCR was used for relative quantitation by targeting the 18S region of the rRNA gene, total bacterial abundance by targeting the 16S region of the rRNA gene (22), and the abundance of key bacterial species, including the four most common vaginal *Lactobacillus* species (*L. crispatus, L. iners, L. jensenii,* and *L. gasseri*) (23) and four common BV-associated bacterial taxa [*G. vaginalis, Atopobium vaginae, Megasphaera* species (24), and *Prevotella* species (25)]. All semi-quantitative PCR assays were Taqman-based and performed on the QuantStudio 6 Flex Real-Time PCR System (Thermofisher). Primer and probe sequences are presented in Table S1. Total reaction volume for assays was 10 µL. Assays for total fungal and bacterial load, *Prevotella*, *L. crispatus, L. iners, L. jensenii*, and *L. gasseri* were performed at 95°C for 10 min, 45 cycles at 95°C for 15 s, then 60°C for 1 min. Assays for *G. vaginalis, A. vaginae*, and *Megasphaera* spp. were performed at 95°C for 10 min, 45 cycles at 95°C for 15 s, then 55°C for 1 min. Data analysis was performed with QuantStudio Real-Time PCR Software version 1.3 (Applied Biosystems). Copy numbers were quantified using the ΔCt method as described in the following equation, where ΔCt represents the difference in Ct between the negative control and sample: Copy number = 2^ΔCt. This method assumes 100% reaction efficiency. To compare the 2^ΔCt method to other quantitative PCR methods, we also measured a subset of key vaginal bacteria (*L. crispatus, L. iners,* and *G. vaginalis*) at the pre- and post-metronidazole visits using the standard curve method (26). For generation of standard curves, *L. crispatus* (ATCC, 33820) was grown in chopped meat media (Anaerobic Systems) anaerobically (80% N_2_; 10% CO_2_; 10 H_2_) at 37°C; *L. iners* (ATCC, 55195) and *G. vaginalis* (ATCC, 14019) were grown in New York City III media aerobically at 37°C and anaerobically at 37°C, respectively. DNA was extracted from bacterial cultures using the same method as described above and serially diluted 1 in 10 five times for the generation of standard curves. Comparison of semi-quantitative (2^ΔCt method) and quantitative (standard curve method) PCR results are presented in Fig. S8.

### Soluble immune factor measurement

Cervicovaginal swab eluents were thawed and centrifuged at 4,500 rpm for 30 min. Supernatant was then removed for immune factor analysis and the bacterial pellet was left intact for semi-quantitative PCR analyses. The soluble immune factors IL-1α, IFN-α2A, IL-17A, IL-6, IP-10, IL-8, macrophage inflammatory protein (MIP) 1β, MIP-3α, MIG, sE-cad, and MMP-9 were measured in duplicate on the Meso Scale Discovery (MSD) platform according to manufacturer’s instructions as previously described (Meso Scale Discovery, Rockville, MD) (27).

### DNA extraction and metagenomic sequencing

Samples were transferred from swab collection tubes into ZymoBIOMICS lysis solution for DNA extraction. DNA was extracted and processed with high-throughput automation liquid handlers (Agilent Bravo system and Labcyte ECHO instrument) to maintain constancy in sample experimentation and decrease laboratory processing time. The ZymoBIOMICS 96 MagBead DNA kit was followed as instructed to extract DNA from swab samples, water controls, and storage medium controls. Illumina library preparation was performed using a miniaturized protocol of NEBNext Ultra II FS DNA Library Prep kit for DNA for the Labcyte ECHO instrument13. More than 25 million paired-end 150 bp reads per patient sample were collected on an Illumina NovaSeq instrument. The CZ ID platform was used to process raw sequencing reads and remove host reads using default parameters (28). Samples with more than 100,000 reads were included in the present analyses to filter out low-quality samples. The VIRGO bioinformatic pipeline was used to align processed metagenomic reads with established databases to identify bacterial taxa at the species level (29). Kraken2 v.2.1.2 (30) was used in combination with Pavian v.1.0 (31) to taxonomically classify fungal reads using default parameters and a confidence scoring threshold of 0.5 using the complete RefSeq database of fungal genomes.

### Statistical analysis

The primary analysis of this study assessed the change in vaginal fungal abundance immediately following metronidazole treatment and subsequent analyses were hypothesis generating. Soluble immune factor levels and gene copy numbers were normalized through log_10_ transformation. Continuous variables were compared with Mann-Whitney U-test (if unpaired) and the Wilcoxon matched pairs signed-rank test (if paired). Categorical variables were compared with Pearson’s χ^2^ test (if unpaired) and the McNemar test (if paired). Association between continuous variables was determined with linear regression. Linear mixed models were generated to evaluate the association between paired measurements without violating independence using the nlme package in R. Shannon diversity and ANOSIM of the vaginal microbiota were calculated using the vegan package in R. Where relevant, *P*-values were adjusted for multiple comparisons with the FDR using the “FDR” command in the fuzzySim R package. All statistical tests were performed in GraphPad Prism or RStudio.

## Data Availability

Metagenomic data are available from the NCBI Sequence Read Archive under BioProject ID PRJNA784288 and PRJNA1090602. All other data and data analysis scripts are available upon request from the corresponding author.

## SUPPLEMENTAL MATERIAL

Supplemental figures and tables spectrum.03501-23-s0001.docx Fig. S1-S8; Tables S1-S7.

ASM does not own the copyrights to Supplemental Material that may be linked to, or accessed through, an article. The authors have granted ASM a non-exclusive, world-wide license to publish the Supplemental Material files. Please contact the corresponding author directly for reuse.

## References

[1] Abou Chacra L, Fenollar F. 2021. Exploring the global vaginal microbiome and its impact on human health. Microb Pathog 160:105172. doi:10.1016/j.micpath.2021.105172 34500016

[2] Ravel J, Gajer P, Abdo Z, Schneider GM, Koenig SSK, McCulle SL, Karlebach S, Gorle R, Russell J, Tacket CO, Brotman RM, Davis CC, Ault K, Peralta L, Forney LJ. 2011. Vaginal microbiome of reproductive-age women. Proc Natl Acad Sci U S A 108:4680–4687. doi:10.1073/pnas.1002611107 20534435 PMC3063603

[3] Bradford LL, Ravel J. 2017. The vaginal mycobiome: a contemporary perspective on fungi in women’s health and diseases. Virulence 8:342–351. doi:10.1080/21505594.2016.1237332 27657355 PMC5411243

[4] Sobel JD. 1985. Epidemiology and pathogenesis of recurrent vulvovaginal candidiasis. Am J Obstet Gynecol 152:924–935. doi:10.1016/s0002-9378(85)80003-x 3895958

[5] van de Wijgert J, Morrison CS, Cornelisse PGA, Munjoma M, Moncada J, Awio P, Wang J, Van der Pol B, Chipato T, Salata RA, Padian NS. 2008. Bacterial vaginosis and vaginal yeast, but not vaginal cleansing, increase HIV-1 acquisition in African women. J Acquir Immune Defic Syndr 48:203–210. doi:10.1097/QAI.0b013e3181743936 18520679

[6] Farr A, Kiss H, Holzer I, Husslein P, Hagmann M, Petricevic L. 2015. Effect of asymptomatic vaginal colonization with Candida albicans on pregnancy outcome. Acta Obstet Gynecol Scand 94:989–996. doi:10.1111/aogs.12697 26084843

[7] Brown SE, Schwartz JA, Robinson CK, OʼHanlon DE, Bradford LL, He X, Mark KS, Bruno VM, Ravel J, Brotman RM. 2019. The vaginal microbiota and behavioral factors associated with genital Candida albicans detection in reproductive-age women. Sex Transm Dis 46:753–758. doi:10.1097/OLQ.0000000000001066 31517769 PMC6818707

[8] McClelland RS, Richardson BA, Hassan WM, Graham SM, Kiarie J, Baeten JM, Mandaliya K, Jaoko W, Ndinya-Achola JO, Holmes KK. 2009. Prospective study of vaginal bacteria flora and other risk factors for vulvovaginal candidiasis. J Infect Dis 199:1883–1890. doi:10.1086/599213 19456235 PMC2743896

[9] Shukla A, Sobel JD. 2019. Vulvovaginitis caused by Candida species following antibiotic exposure. Curr Infect Dis Rep 21:44. doi:10.1007/s11908-019-0700-y 31707496

[10] Pirotta MV, Garland SM. 2006. Genital Candida species detected in samples from women in Melbourne, Australia, before and after treatment with antibiotics. J Clin Microbiol 44:3213–3217. doi:10.1128/JCM.00218-06 16954250 PMC1594690

[11] Cohen CR, Wierzbicki MR, French AL, Morris S, Newmann S, Reno H, Green L, Miller S, Powell J, Parks T, Hemmerling A. 2020. Randomized trial of Lactin-V to prevent recurrence of bacterial vaginosis. N Engl J Med 382:1906–1915. doi:10.1056/NEJMoa1915254 32402161 PMC7362958

[12] Armstrong E, Hemmerling A, Miller S, Burke KE, Newmann SJ, Morris SR, Reno H, Huibner S, Kulikova M, Liu R, Crawford ED, Castañeda GR, Nagelkerke N, Coburn B, Cohen CR, Kaul R. 2022. Metronidazole treatment rapidly reduces genital inflammation through effects on bacterial vaginosis-associated bacteria rather than lactobacilli. J Clin Invest 132:e152930. doi:10.1172/JCI152930 35113809 PMC8920324

[13] Mengesha BG, Conti HR. 2017. The role of IL-17 in protection against mucosal Candida infections. J Fungi (Basel) 3:52. doi:10.3390/jof3040052 29371568 PMC5753154

[14] Ballou ER, Avelar GM, Childers DS, Mackie J, Bain JM, Wagener J, Kastora SL, Panea MD, Hardison SE, Walker LA, Erwig LP, Munro CA, Gow NAR, Brown GD, MacCallum DM, Brown AJP. 2016. Lactate signalling regulates fungal β-glucan masking and immune evasion. Nat Microbiol 2:16238. doi:10.1038/nmicrobiol.2016.238 27941860 PMC5704895

[15] Ene IV, Cheng S-C, Netea MG, Brown AJP. 2013. Growth of Candida albicans cells on the physiologically relevant carbon source lactate affects their recognition and phagocytosis by immune cells. Infect Immun 81:238–248. doi:10.1128/IAI.01092-12 23115042 PMC3536122

[16] Glocker E-O, Hennigs A, Nabavi M, Schäffer AA, Woellner C, Salzer U, Pfeifer D, Veelken H, Warnatz K, Tahami F, Jamal S, Manguiat A, Rezaei N, Amirzargar AA, Plebani A, Hannesschläger N, Gross O, Ruland J, Grimbacher B. 2009. A homozygous CARD9 mutation in a family with susceptibility to fungal infections. N Engl J Med 361:1727–1735. doi:10.1056/NEJMoa0810719 19864672 PMC2793117

[17] Eyerich K, Foerster S, Rombold S, Seidl HP, Behrendt H, Hofmann H, Ring J, Traidl-Hoffmann C. 2008. Patients with chronic mucocutaneous candidiasis exhibit reduced production of Th17-associated cytokines IL-17 and IL-22. J Invest Dermatol 128:2640–2645. doi:10.1038/jid.2008.139 18615114

[18] Davidson L, van den Reek J, Bruno M, van Hunsel F, Herings RMC, Matzaraki V, Boahen CK, Kumar V, Groenewoud HMM, van de Veerdonk FL, Netea MG, de Jong E, Kullberg BJ. 2022. Risk of candidiasis associated with interleukin-17 inhibitors: a real-world observational study of multiple independent sources. Lancet Reg Health Eur 13:100266. doi:10.1016/j.lanepe.2021.100266 34950923 PMC8671639

[19] Armstrong E, Hemmerling A, Miller S, Burke KE, Newmann SJ, Morris SR, Reno H, Huibner S, Kulikova M, Nagelkerke N, Coburn B, Cohen CR, Kaul R. 2022. Sustained effect of LACTIN-V (Lactobacillus crispatus CTV-05) on genital immunology following standard bacterial vaginosis treatment: results from a randomised, placebo-controlled trial. Lancet Microbe 3:e435–e442. doi:10.1016/S2666-5247(22)00043-X 35659905 PMC9188188

[20] MacAlpine J, Daniel-Ivad M, Liu Z, Yano J, Revie NM, Todd RT, Stogios PJ, Sanchez H, O’Meara TR, Tompkins TA, Savchenko A, Selmecki A, Veri AO, Andes DR, Fidel PL, Robbins N, Nodwell J, Whitesell L, Cowen LE. 2021. A small molecule produced by Lactobacillus species blocks Candida albicans filamentation by inhibiting a DYRK1-family kinase. Nat Commun 12:6151. doi:10.1038/s41467-021-26390-w 34686660 PMC8536679

[21] Alonso-Roman R, Last A, Mirhakkak MH, Sprague JL, Möller L, Großmann P, Graf K, Gratz R, Mogavero S, Vylkova S, Panagiotou G, Schäuble S, Hube B, Gresnigt MS. 2022. Lactobacillus rhamnosus colonisation antagonizes Candida albicans by forcing metabolic adaptations that compromise pathogenicity. Nat Commun 13:3192. doi:10.1038/s41467-022-30661-5 35680868 PMC9184479

[22] Liu CM, Kachur S, Dwan MG, Abraham AG, Aziz M, Hsueh PR, Huang YT, Busch JD, Lamit LJ, Gehring CA, Keim P, Price LB. 2012. FungiQuant: a broad-coverage fungal quantitative real-time PCR assay. BMC Microbiol 12:255. doi:10.1186/1471-2180-12-255 23136846 PMC3565980

[23] Balashov SV, Mordechai E, Adelson ME, Sobel JD, Gygax SE. 2014. Multiplex quantitative polymerase chain reaction assay for the identification and quantitation of major vaginal lactobacilli. Diagn Microbiol Infect Dis 78:321–327. doi:10.1016/j.diagmicrobio.2013.08.004 24445159

[24] Kusters JG, Reuland EA, Bouter S, Koenig P, Dorigo-Zetsma JW. 2015. A multiplex real-time PCR assay for routine diagnosis of bacterial vaginosis. Eur J Clin Microbiol Infect Dis 34:1779–1785. doi:10.1007/s10096-015-2412-z 26143346 PMC4545173

[25] Martin FE, Nadkarni MA, Jacques NA, Hunter N. 2002. Quantitative microbiological study of human carious dentine by culture and real-time PCR: association of anaerobes with histopathological changes in chronic pulpitis. J Clin Microbiol 40:1698–1704. doi:10.1128/JCM.40.5.1698-1704.2002 11980945 PMC130955

[26] Larionov A, Krause A, Miller WR. 2005. A standard curve based method for relative real time PCR data processing. BMC Bioinformatics 6:62. doi:10.1186/1471-2105-6-62 15780134 PMC1274258

[27] Mohammadi A, Bagherichimeh S, Perry MC, Fazel A, Tevlin E, Huibner S, Tharao W, Coburn B, Kaul R. 2020. The impact of cervical cytobrush sampling on cervico-vaginal immune parameters and microbiota relevant to HIV susceptibility. Sci Rep 10:8514. doi:10.1038/s41598-020-65544-6 32444843 PMC7244754

[28] Kalantar KL, Carvalho T, de Bourcy CFA, Dimitrov B, Dingle G, Egger R, Han J, Holmes OB, Juan Y-F, King R, et al.. 2020. IDseq-an open source cloud-based pipeline and analysis service for metagenomic pathogen detection and monitoring. Gigascience 9:giaa111. doi:10.1093/gigascience/giaa111 33057676 PMC7566497

[29] Ma B, France MT, Crabtree J, Holm JB, Humphrys MS, Brotman RM, Ravel J. 2020. A comprehensive non-redundant gene catalog reveals extensive within-community intraspecies diversity in the human vagina. Nat Commun 11:940. doi:10.1038/s41467-020-14677-3 32103005 PMC7044274

[30] Wood DE, Lu J, Langmead B. 2019. Improved metagenomic analysis with Kraken 2. Genome Biol 20:257. doi:10.1186/s13059-019-1891-0 31779668 PMC6883579

[31] Breitwieser FP, Salzberg SL. 2020. Pavian: interactive analysis of metagenomics data for microbiome studies and pathogen identification. Bioinformatics 36:1303–1304. doi:10.1093/bioinformatics/btz715 31553437 PMC8215911

