# Supplementary material for: Vaginal fungi are associated with treatment-induced shifts in the vaginal microbiota and with a distinct genital immune profile
Source: Microbiol Spectr. 2024 Jun 24;12(8):e03501-23. doi: 10.1128/spectrum.03501-23 (PMC11302301; doi:10.1128/spectrum.03501-23)
Supplement: Supplemental figures and tables — Fig. S1-S8; Tables S1-S7. [file spectrum.03501-23-s0001.docx]

**SUPPLEMENTAL MATERIAL**

**FIGURES**


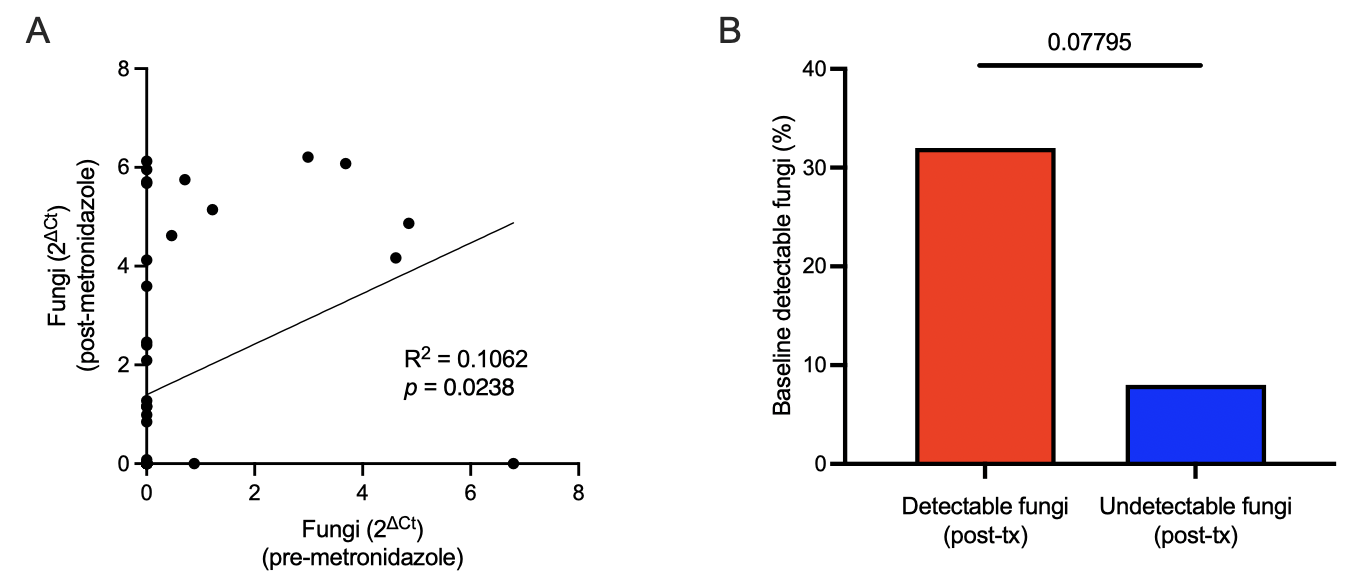


**Supplemental figure S1:** **Pre-treatment vaginal fungi as a predictor of post-treatment fungal expansion.** A) Association between pre- and post-treatment fungal relative abundance by semi-quantitative PCR. B) Comparison of the proportion of women with detectable fungi post-treatment by semi-quantitative PCR based on pre-treatment detectability. *P* values determined with linear regression or Pearson Chi-square test.


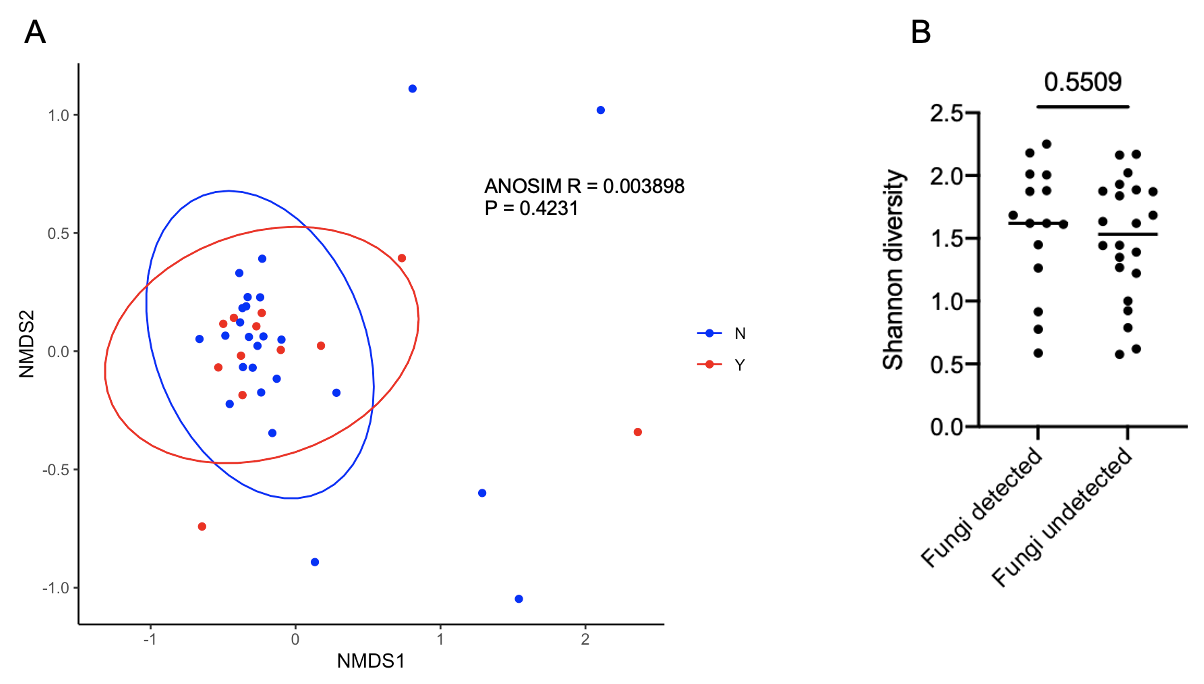


**Supplemental figure S2:** **Pre-treatment vaginal microbiota composition as a predictor of fungal detectability post-treatment.** A) Non-metric multidimensional scaling (NMDS) plot constructed with species-level Bray-Curtis dissimilarities of each participant prior to metronidazole treatment. B) Shannon diversity of the vaginal microbiota prior to metronidazole treatment based on fungal detectability immediately following treatment. *P* values determined with ANOSIM or Mann-Whitney U test.


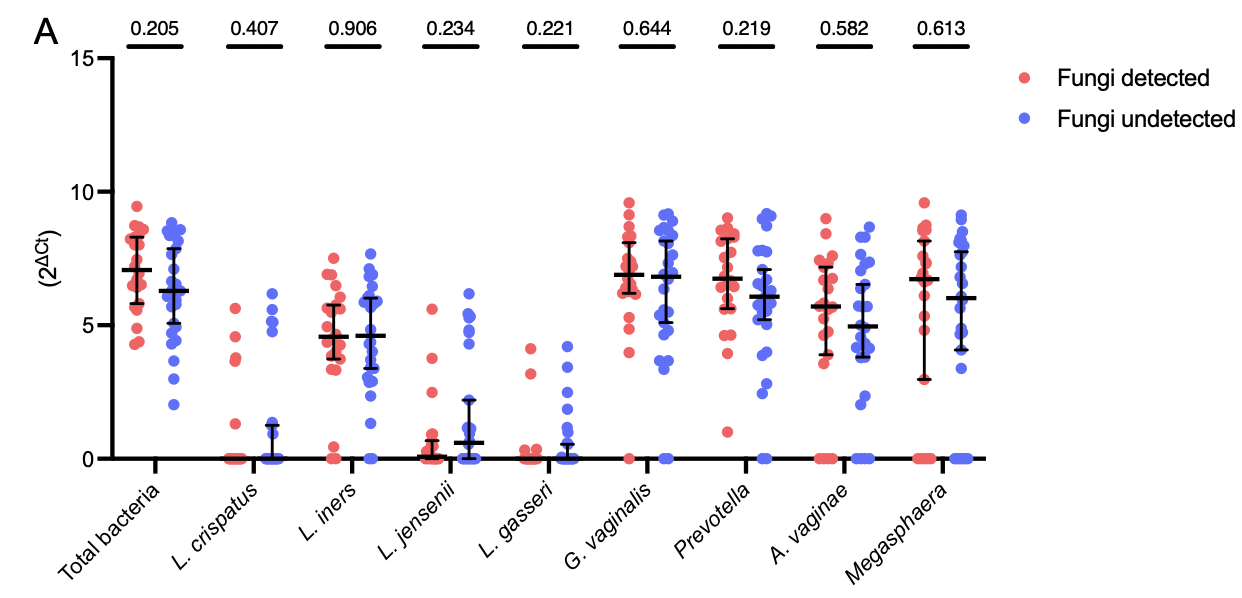


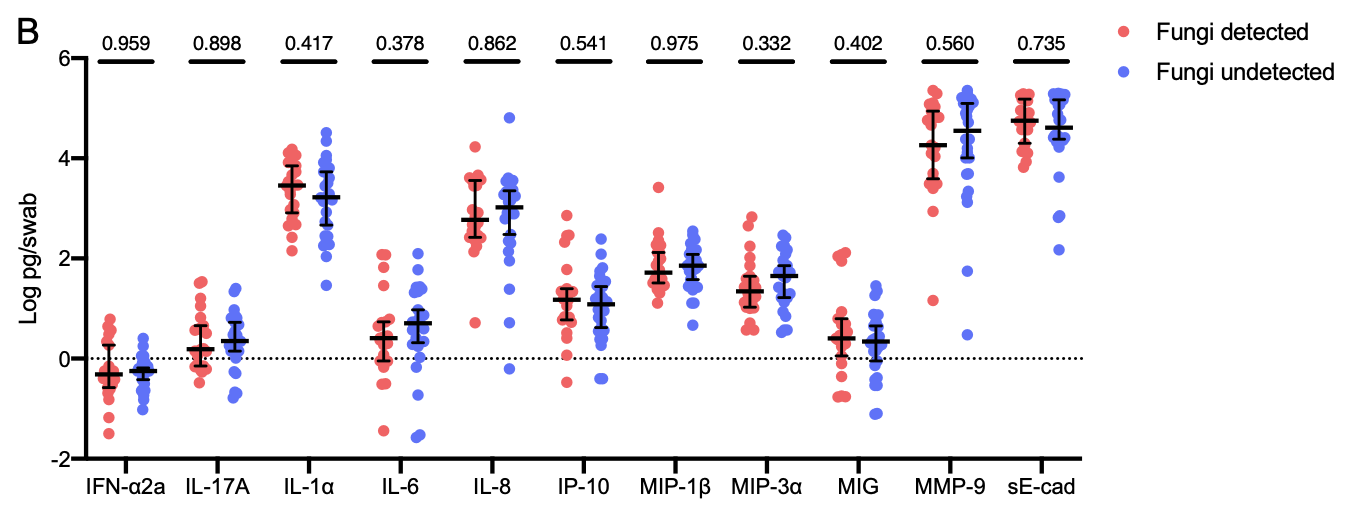


**Supplemental figure S3:** **Pre-metronidazole microbial and immune predictors of detectable fungi immediately following metronidazole treatment.** Comparison of A) relative bacterial abundance and relative abundance of *L. crispatus*, *L. iners*, *L. jensenii*, *L. gasseri*, *G. vaginalis*, *Prevotella*, *A. vaginae*, and *Megasphaera* immediately prior to metronidazole treatment between women with detectable and undetectable fungi immediately following treatment. B) Comparison of IFN-α2A, IL-17A, IL-1α, IL-6, IL-8, IP-10, MIP-1β, MIP-3α, MIG, MMP-9, and sE-cad levels prior to metronidazole treatment between women with detectable fungi after metronidazole treatment. *P* values determined with Mann-Whitney U test.


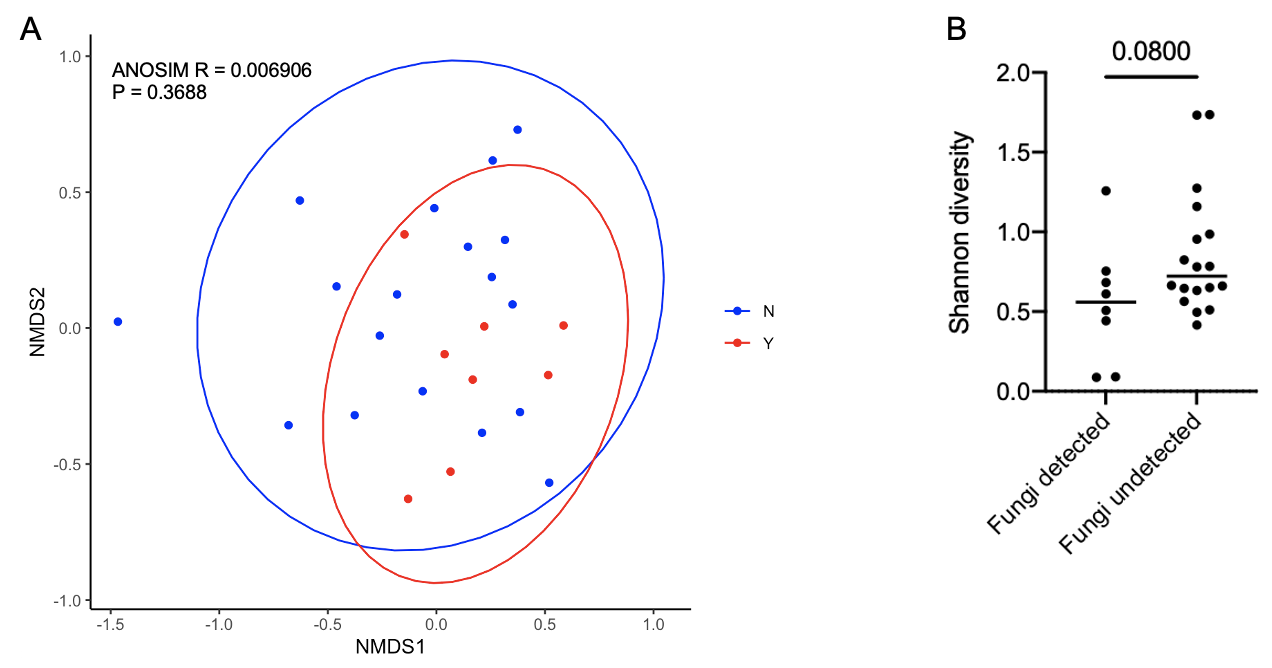


**Supplemental figure S4:** **Post-treatment vaginal microbiota composition as a correlate of fungal detectability post-treatment.** A) Non-metric multidimensional scaling (NMDS) plot constructed with species-level Bray-Curtis dissimilarities of each participant immediately following metronidazole treatment. B) Shannon diversity of the vaginal microbiota immediately following metronidazole treatment based on fungal detectability immediately following treatment. *P* values determined with ANOSIM or Mann-Whitney U test.


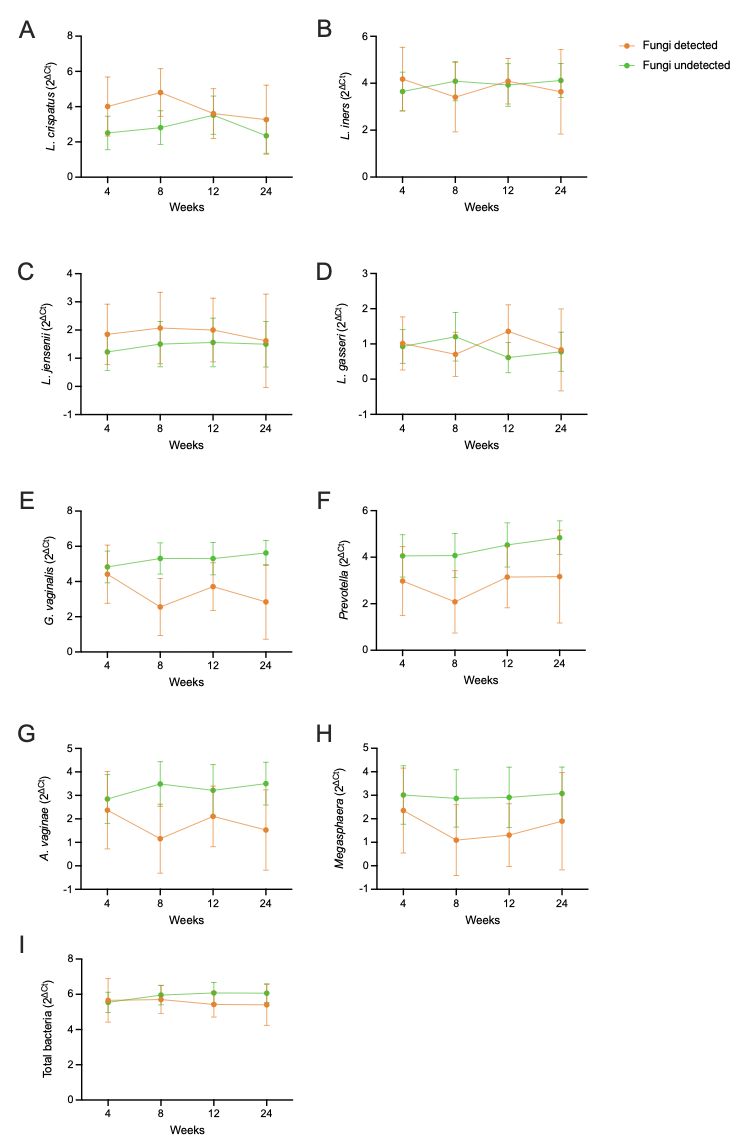


**Supplemental figure S5: Longitudinal dynamics of vaginal bacteria based on fungal detectability during and after study product administration.** Relative abundance of A) *L. crispatus*, B) *L. iners*, C) *L. jensenii*, D) *L. gasseri*, E) *G. vaginalis*, F) *Prevotella* spp., G) *A. vaginae*, H) *Megasphaera* spp., and I) total bacteria by semi-quantitative PCR based on fungal detectability at each visit during and after study product administration.


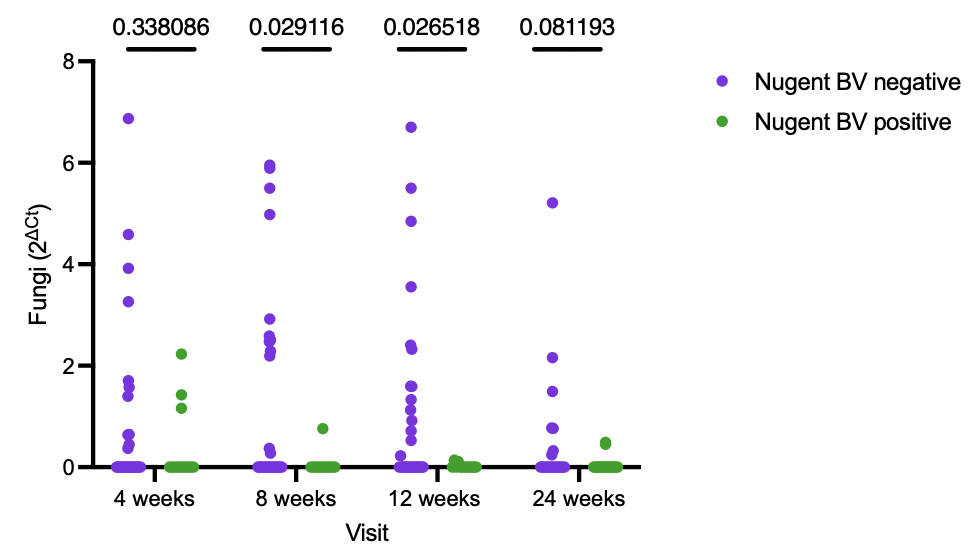


**Supplemental figure S6:** **Fungal relative abundance during study product administration based on Nugent BV.** BV defined as Nugent score of 7 or greater. All participants diagnosed with a yeast infection at any timepoint excluded. *P* values determined with Mann-Whitney U test.

**
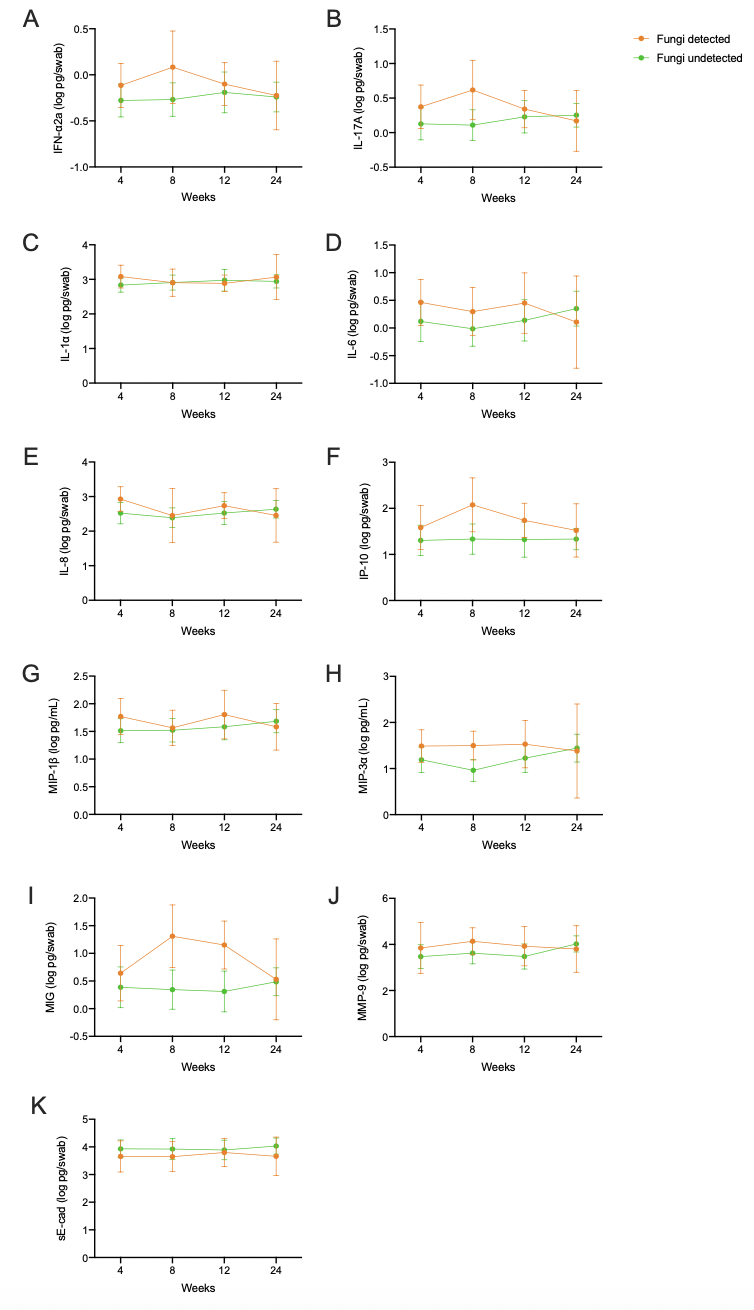
**

**Supplemental figure S7: Longitudinal dynamics of vaginal soluble immune factors based on fungal detectability during and after study product administration.** Vaginal levels of A) IFN-α2a, B) IL-17A, C) IL-1α, D) IL-6, E) IL-8, F) IP-10, G) MIP-1β, H) MIP-3α, I) MIG, J) MMP-9, and K) sE-cad during and after study product administration based on fungal detectability.


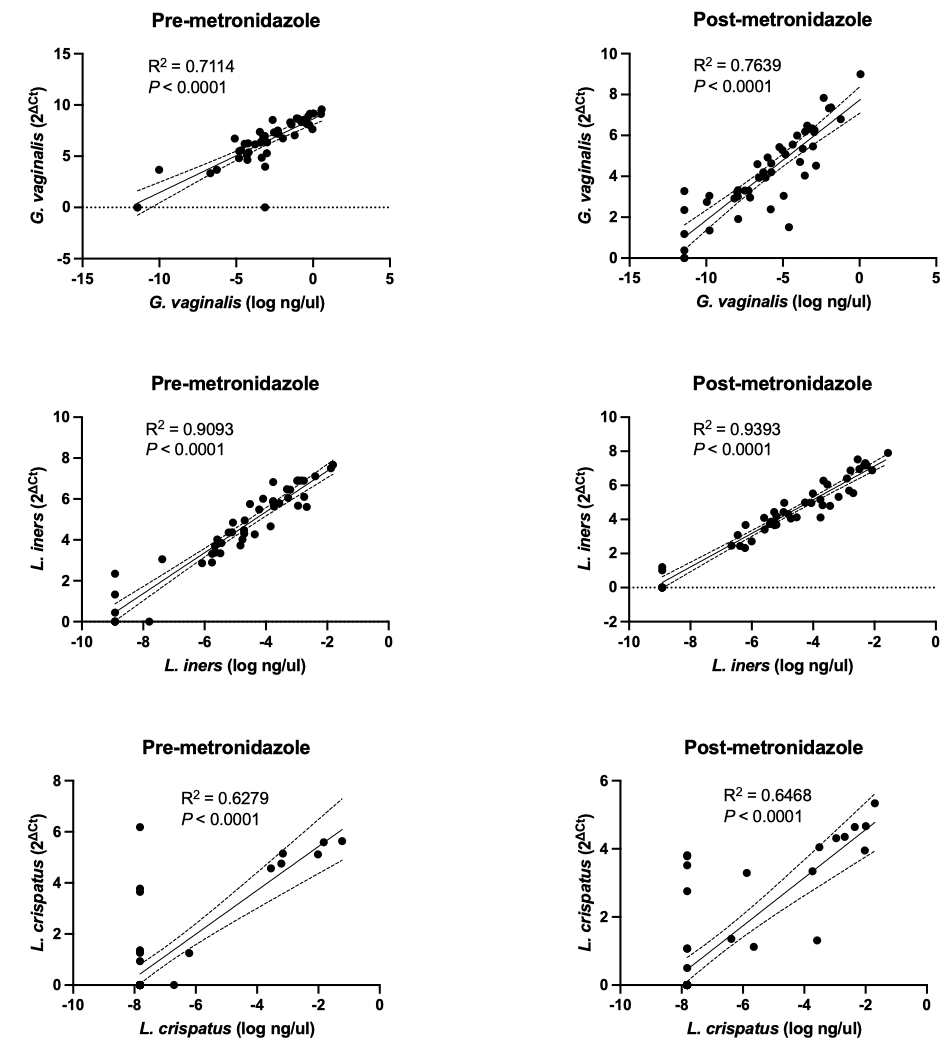


**Supplemental figure S8: Comparison of bacterial abundances with qPCR using the 2^ΔCt and standard curve methods.** Vaginal abundances of *G. vaginalis, L. iners,* and *L. crispatus* at the pre- and post-metronidazole visits were measured with qPCR and compared based on semi-quantitative (2^ΔCt) or quantitative (standard curve) methods. Regression line, 95% confidence bands, *p* values, and R^2^ values determined with linear regression.

**TABLES**

| **Target** | **Oligo** | **Sequence** |
| --- | --- | --- |
| 18S | Forward | GGRAAACTCACCAGGTCCAG |
|  | Reverse | GSWCTATCCCCAKCACGA |
|  | Probe | [Cy5]-TGGTGCATGGCCGTT-[NFQ] |
| 16S | Forward | 5-TCCTACGGGAGGCAGCAGT-3 |
|  | Reverse | 5-GGACTACCAGGGTATCTAATCCTGTT-3 |
|  | Probe | (FAM)-CGTATTACCGCGGCTGCTGGCAC-(NFQ-MGB) |
| *L. crispatus* | Forward | CGTGGTTCAGCWTTGAAGGC |
|  | Reverse | CTTCAACTGGCATYAAGAATGGC |
|  | Probe | [ROX]-AGGCGACAAGGAAGCTCAAGAAC-BHQ2 |
| *L. iners* | Forward | CGTGGTTCAGCWTTGAAGGC |
|  | Reverse | CTTCAACTGGCATYAAGAATGGC |
|  | Probe | [HEX]-AGGCGATCCAGAACAAGAAGCAG-BHQ1 |
| *L. gasseri* | Forward | CGTGGTTCAGCWTTGAAGGC |
|  | Reverse | CTTCAACTGGCATYAAGAATGGC |
|  | Probe | [FAM]-AGGTGACCCAGAACAACAAGACG-BHQ1 |
| *L. jensenii* | Forward | CGTGGTTCAGCWTTGAAGGC |
|  | Reverse | CTTCAACTGGCATYAAGAATGGC |
|  | Probe | [Cy5]-AGGTGACCCAGAACAAGAAAAGGT-BHQ2 |
| *G. vaginalis* | Forward | GCGGGCTAGAGTGCA |
|  | Reverse | ACCCGTGGAATGGGCC |
|  | Probe | [ROX]CTTCTCAGCGTCAGTAACAGC |
| *A. vaginae* | Forward | TAGGTCAGGAGTTAAATCTG |
|  | Reverse | TCATGGCCCAGAAGACCGCC |
|  | Probe | [HEX]CTACCAGACTCAAGCCTGCC |
| *Megasphaera* | Forward | GATGCCAACAGTATCCGTCCG |
|  | Reverse | CCTCTCCGACACTCAAGTTCGA |
|  | Probe | [FAM]ACAGACTTACCGAACCGCCT |
| *Prevotella* | Forward | 5-CCAGCCAAGTAGCGTGCA-3 |
|  | Reverse | 5-TGGACCTTCCGTATTACCGC-3 |
|  | Probe | (56-FAM)-AATAAGGACCGGCTAATTCCGTGCCAG-(36-TAMSp) |

**Supplementary table S1.** **Primer and probe sequences for semi-quantitative polymerase chain reaction.**

| **Pre-metronidazole** | | | |
| --- | --- | --- | --- |
| **Semi-quantitative PCR** | **Metagenomics** | |  |
|  | Detectable | Undetectable | **Total** |
| Detectable | 4 | 3 | 7 |
| Undetectable | 3 | 27 | 30 |
| **Total** | 7 | 30 | 37 |
| **Post-metronidazole** | | | |
| **Semi-quantitative PCR** | **Metagenomics** | |  |
|  | Detectable | Undetectable | **Total** |
| Detectable | 6 | 2 | 8 |
| Undetectable | 2 | 16 | 18 |
| **Total** | 8 | 18 | 26 |

**Supplementary table S2.** Concordance in fungal detectability between semi-quantitative PCR and metagenomic sequencing for pre- and post-metronidazole visits.

|  | **Detectable fungi**  **(n=22)** | **Undetectable fungi**  **(n=26)** | **P value** |
| --- | --- | --- | --- |
| **Age** | 32.0 (30.0, 36.5) | 29.5 (24.0, 35.0) | 0.3197 |
| **Race** |  |  |  |
| Asian | 2/22 (9%) | 1/26 (4%) | 0.8811 |
| Black or African American | 9/22 (41%) | 11/26 (42%) | 1 |
| Multiracial | 0/22 (0%) | 1/26 (4%) | 1 |
| White | 11/22 (50%) | 9/26 (35%) | 0.4334 |
| Unstated | 0/22 (0%) | 4/26 (15%) | 0.1623 |
| **Sex between pre- and post-treatment** | 5/22 (23%) | 8/26 (31%) | 0.7651 |
| **Contraceptive use** |  |  |  |
| Injectable | 1/22 (5%) | 0/26 (0%) | 0.9327 |
| Oral contraceptive | 2/22 (9%) | 2/26 (8%) | 1 |
| Implant | 0/22 (0%) | 1/26 (4%) | 1 |
| Hormonal IUD | 1/22 (5%) | 3/26 (12%) | 0.7268 |
| Contraceptive patch | 1/22 (5%) | 0/26 (0%) | 0.9327 |
| None | 17/22 (77%) | 19/26 (73%) | 1 |
| Data are median (interquartile range) or frequency (percent).  *P* values determined with Mann-Whitney U test for continuous variables and Pearson Chi-square test for proportions. | | | |

**Supplementary table S3.** Sociodemographic characteristics and sexual activity among participants with detectable and undetectable fungi following metronidazole treatment.

|  | **Controlling for treatment group** | |
| --- | --- | --- |
|  | **Linear mixed model coefficient** | **P value** |
| **Total bacteria** | -0.07 | 0.2406 |
| ***L. crispatus*** | 0.31 | 0.0043 |
| ***L. iners*** | 0.16 | 0.473 |
| ***L. jensenii*** | 0.26 | 0.0024 |
| ***L. gasseri*** | 0.04 | 0.5952 |
| ***G. vaginalis*** | -0.36 | 0.0014 |
| ***Prevotella*** | -0.40 | 0.0006 |
| ***A. vaginae*** | -0.44 | 0.0003 |
| ***Megasphaera*** | -0.41 | 0.0024 |
| *P* values determined with linear mixed models including measurements obtained during and after study product administration with total fungal relative abundance as the independent variable.  All participants diagnosed with a yeast infection at any timepoint were excluded.  *P* values < 0.05 highlighted. | | |

**Supplementary table S4.** Association between fungal abundance and bacterial relative abundances by semi-quantitative PCR during and after study product administration while controlling for treatment group.

|  | **Controlling for treatment group** | |
| --- | --- | --- |
|  | **Linear mixed model coefficient** | **P value** |
| **IFN-a2a** | 0.08 | 0.0015 |
| **IL-17A** | 0.10 | 0.0011 |
| **IL-1a** | 0.01 | 0.7078 |
| **IL-6** | 0.08 | 0.1003 |
| **IL-8** | 0.00 | 0.9868 |
| **IP-10** | 0.18 | <0.0001 |
| **MIP-1b** | 0.03 | 0.2918 |
| **MIP-3a** | 0.06 | 0.1441 |
| **MIG** | 0.17 | <0.0001 |
| **MMP-9** | -0.09 | 0.1627 |
| **sE-cad** | -0.08 | 0.0816 |
| *P* values determined with linear mixed models including measurements obtained during and after study product administration with total fungal relative abundance by semi-quantitative PCR as the independent variable.  All participants diagnosed with a yeast infection excluded.  *P* values < 0.05 highlighted. | | |

**Supplementary table S5.** Association between fungal abundance and immune factors during and after study product administration while controlling for treatment group.

|  | **Controlling for *Lactobacillus* species** | | **Controlling for BV-associated bacteria** | |
| --- | --- | --- | --- | --- |
|  | **Linear mixed model coefficient** | **P value** | **Linear mixed model coefficient** | **P value** |
| **IFN-a2a** | 0.07 | 0.046 | 0.07 | 0.0055 |
| **IL-17A** | 0.09 | 0.0020 | 0.11 | 0.0003 |
| **IL-1a** | -0.01 | 0.8600 | 0.04 | 0.1389 |
| **IL-6** | 0.07 | 0.1280 | 0.11 | 0.0145 |
| **IL-8** | -0.02 | 0.6936 | 0.02 | 0.5392 |
| **IP-10** | 0.16 | 0.0001 | 0.16 | 0.0001 |
| **MIP-1b** | 0.03 | 0.3155 | 0.05 | 0.0898 |
| **MIP-3a** | 0.06 | 0.1436 | 0.07 | 0.0608 |
| **MIG** | 0.14 | 0.0009 | 0.17 | 0.0001 |
| **MMP-9** | -0.14 | 0.0367 | -0.04 | 0.5174 |
| **sE-cad** | -0.09 | 0.0433 | 0.02 | 0.5261 |
| *P* values determined with linear mixed model including measurements obtained during and after study product administration with fungal relative abundance by semi-quantitative PCR as the independent variable.  All participants diagnosed with a yeast infection excluded.  *P* values < 0.05 highlighted. | | | | |

**Supplementary table S6.** Association between fungal relative abundance by semi-quantitative PCR and immune factors during and after study product administration while controlling for bacterial abundances.

|  | **Controlling for *Lactobacillus* species and treatment group** | | **Controlling for BV-associated bacteria and treatment group** | |
| --- | --- | --- | --- | --- |
|  | **Linear mixed model coefficient** | **P value** | **Linear mixed model coefficient** | **P value** |
| **IFN-a2a** | 0.07 | 0.0035 | 0.07 | 0.0043 |
| **IL-17A** | 0.09 | 0.0022 | 0.12 | 0.0003 |
| **IL-1a** | -0.01 | 0.8164 | 0.04 | 0.1501 |
| **IL-6** | 0.07 | 0.1329 | 0.11 | 0.0146 |
| **IL-8** | -0.02 | 0.6978 | 0.03 | 0.5226 |
| **IP-10** | 0.16 | 0.0001 | 0.16 | 0.0001 |
| **MIP-1b** | 0.03 | 0.3043 | 0.05 | 0.0834 |
| **MIP-3a** | 0.06 | 0.1354 | 0.07 | 0.0562 |
| **MIG** | 0.14 | 0.0010 | 0.17 | 0.0001 |
| **MMP-9** | -0.14 | 0.0329 | -0.04 | 0.5068 |
| **sE-cad** | -0.09 | 0.0372 | 0.02 | 0.5530 |
| *P* values determined with linear mixed model including measurements obtained during and after study product administration with fungal relative abundance by semi-quantitative PCR as the independent variable.  All participants diagnosed with a yeast infection excluded.  *P* values < 0.05 highlighted. | | | | |

**Supplementary table S7.** Association between fungal relative abundance by semi-quantitative PCR and immune factors during and after study product administration while controlling for bacterial abundances and treatment group.
